# The Thymus Treatment of Inoperable Cancer

**Published:** 1911-01-21

**Authors:** 


### The Thymus Treatment of Inoperable Cancer.

There can be no doubt that the only curative treatment of cancer is by surgical removal of the tumour in its early stages. Unfortunately, however, many cases come under observation when this curable stage has passed and surgery can be but palliative. The question that arises is, What other measures can be adopted to mitigate the patient's distress? A very interesting account of surgery in Japan has recently been published in a contemporary by Professor Yoshihiro Takaki, in which he gives his experiences. He finds little or no benefit from Coley's fluid, zinc chloride paste, trypsin, or thyroid extract, but, without going into any extravagances as to the results he has obtained, he is convinced that great relief. particularly from the pain, can be attained by the use of thymus extract in these cases. This line of treatment is due to Dr. Frederick Gwyer, of New York, whose original paper will be found in the *Annals of Surgery*, 1907. The practical results obtained by Dr. Gwyer himself showed that in cases where the treatment was tried there was marked decrease, or even elimination, of pain, accompanied by a diminution in the size of the cancer masses, whether primary or secondary, together with improvement in digestion and general condition, with more regular action of the bowels, clearer skin and eyes, and a diminished sense of ill-health.

The glands used were received fresh. The fat was removed, and the glandular substance cut up and dried at a low temperature by a forced draught of air, then ground and sifted to a uniform powder. Of this a dose of from 1 to 4 drachms was given three or four times a day. Gwyer also prepared a watery extract from the dried gland by adding the dried powder and some thymol to a solution of normal saline, straining and filtering as rapidly as possible, filtering twice, and then adding 50 per cent. acetic acid. The precipitate so obtained was separated by filtration and redissolved, using about 1½ ounce of the solution. Each drachm of the solution represents the products from half a drachm of the dried gland. The process for the production of an ounce of the extract takes about six hours. This fluid extract is administered either by mouth or hypodermically up to 1 drachm.

Dr. Takaki also makes a powder from fresh calf's thymus gland, which is given by the mouth in doses of from 0.01 to 0.05 gram twice a day between meals. There is a tendency for it to cause indigestion, so that it is best to give quite small doses to begin with, and it is frequently beneficial to give sodium sulphate at the same time. One of the chief effects is diminution in the pain, so that it is particularly in patients who suffer from this symptom severely, as the result of either primary or secondary growths, that thymus treatment is likely to be adopted. Indigestion is liable to occur, and there seems to be a decidedly increased tendency for the tissues of the malignant growth to undergo suppuration and necrosis. Dr. Takaki's cases are published with what seem to be perfect candour and without bias, so that, although the treatment will not bring about radical cure, it seems certainly worth while adopting it in advanced cases, especially when there is pain. So many new methods in the treatment of inoperable cancer cases have now been suggested that it is desirable they should be published as widely as possible, so that the general practitioner, who sees most of these cases in their late stages, may test the efficacy of the newer suggestions. We hope in the course of the year to deal exhaustively with the whole question, and to publish details of all the known methods which are now on their trial in this country and elsewhere, together with a summary of the results which have so far been obtained.

